# Epithelial circulating tumor cells with a heterogeneous phenotype are associated with metastasis in NSCLC

**DOI:** 10.1007/s00432-021-03681-9

**Published:** 2021-07-13

**Authors:** Yujuan Zhang, Yu Men, Jianyang Wang, Puyuan Xing, Jun Zhao, Junling Li, Danfei Xu, Zhouguang Hui, Wei Cui

**Affiliations:** 1 State Key Laboratory of Molecular Oncology, Department of Clinical Laboratory, National Cancer Center/National Clinical Research Center for Cancer/Cancer Hospital, Chinese Academy of Medical Sciences and Peking Union Medical College, Beijing, China; 2 Department of VIP Medical Services & Radiation Oncology, National Cancer Center/National Clinical Research Center for Cancer/Cancer Hospital, Chinese Academy of Medical Sciences and Peking Union Medical College, Beijing, China; 3 Department of Radiation Oncology, National Cancer Center/National Clinical Research Center for Cancer/Cancer Hospital, Chinese Academy of Medical Sciences and Peking Union Medical College, Beijing, China; 4 Department of Medical Oncology, National Cancer Center/National Clinical Research Center for Cancer/Cancer Hospital, Chinese Academy of Medical Sciences and Peking Union Medical College, Beijing, China; 5 Department of Thoracic Surgery, National Cancer Center/National Clinical Research Center for Cancer/Cancer Hospital, Chinese Academy of Medical Sciences and Peking Union Medical College, Beijing, China

**Keywords:** NSCLC, E-CTCs, E/M-CTCs, EMT, Metastasis

## Abstract

**Objectives:**

To analyze the clinical relevance of heterogeneous phenotypes of peripheral circulating tumor cells (CTCs) in non-small cell lung cancer (NSCLC).

**Materials and Methods:**

CTCs in 5 mL venous blood were enriched using the Canpatrol™ CTC technique in 82 NSCLC patients. And then, CTCs were subjected to RNA in situ hybridization with a combination of epithelial (EpCAM and CK8/18/19) and mesenchymal (vimentin and TWIST1) markers.

**Results:**

According to the fluorescent dots, CTCs were classified into three groups, including epithelial CTCs (E-CTC), hybrid epithelial/mesenchymal phenotypes (E/M-CTCs) and mesenchymal CTCs (M-CTCs). In 82 NSCLC cohort, only 2 patients didn’t detect CTCs, the overall CTCs detection rate was 97.5% (80/82). For 60 treatment naïve NSCLC, only one patient didn’t detect CTCs. The median number of total CTCs, hybrid E/M phenotype CTCs, E-CTCs and M-CTCs per 5 mL blood was 22 (range 1–90), 13 (range 0–83), 1 (range 0–17 and 0–47), respectively. Hybrid E/M CTCs, especially the e = m-CTCs, significantly differed between patients with and without distant metastasis. M-CTCs in advanced NSCLC patients were significantly more than the numbers observed in early stage patients. Patients with pure hybrid E/M-CTCs showed a lower proportion in distant metastasis positive cohort compared to negative ones (7% vs 22%), while patients with E + E/M CTCs (20% vs 9%) and E/M + M CTCs (33% vs 20%) showed a higher proportion. CTCs dynamic changes after treatment in 12 advanced NSCLC patients suggested that hybrid E/M-CTCs were related to the primary tumor size at baseline, while M-CTCs may suggest the progression of NSCLC.

**Conclusion:**

We concluded that E-CTCs with a hybrid E/M phenotype are associated to metastasis in therapy-naïve NSCLC patients.

**Supplementary Information:**

The online version contains supplementary material available at 10.1007/s00432-021-03681-9.

## Introduction

The most common form of lung cancer, non-small cell lung cancer (NSCLC), accounts for 85% of lung cancers and is a strong contributor of tumor morbidity and mortality worldwide (Bray et al. 2018). NSCLC patients may undergo surgery in early stages, while others may accept adjuvant chemotherapy and/or adjuvant radiotherapy before/after surgery. However, the rates of 5-year relapse and metastasis in early stage patients are about 20%. To improve the treatment of NSCLC, the pathogenesis of NSCLC must be understood to avoid potential relapse (Chemi et al. 2019).

Circulating tumor cells (CTCs) travel in the blood and derive from the primary tumor, having the capability to go through blood vessels and grow at distant organ sites(Tayoun et al. 2019). However, in the process of distant metastasis, CTCs undergo morphological transformation from proliferative phenotypes to migratory and invasive phenotypes, such as reversible epithelial mesenchymal (EMT) and mesenchymal epithelial transition (MET), also termed as phenotypic plasticity(Tulchinsky et al. 2019). EMT/MET has been reported in many studies and tumor types, including lung cancer (Lindsay et al. 2017; Tayoun et al. 2019). Further studies have reported that CTCs EMT/MET phenotypes are related to patient prognosis. For example, CTCs mesenchymal phenotype in breast cancer patients is associated with distant metastasis (Zhang et al. 2017). However, more recent research showed that the CTCs epithelial phenotype with a restricted mesenchymal transition was strongly associated with lung metastases, while the CTC mesenchymal phenotype showed limited metastatic ability (Liu et al. 2019).

Therefore, here we analyzed CTC phenotypes including epithelial CTCs (E-CTC), hybrid epithelial/mesenchymal CTCs (E/M-CTCs) and mesenchymal CTCs (M-CTCs) in NSCLC to confirm metastasis-related phenotypes using the Canpatrol™ CTCs technique.

## Methods

### Patients and specimens

Between September 2017 and September 2018, 82 lung cancer patients (mean age 59 years, range 35–79 years) at the National Cancer Center/Cancer Hospital, Chinese Academy of Medical Sciences were enrolled and retrospectively analyzed. ﻿Histopathology confirmed the diagnosis for all patients. Among these patients, 60 patients did not receive any treatment and 22 patients had received chemoradiotherapy or targeted therapy. The clinicopathological characteristics of 60 treatment naïve NSCLC is shown in Table 1. 35 out of 60 (58.3%) treatment naïve patients underwent resections of primary tumors. 12/60 (20%) patients received chemoradiotherapy and 13/60 (21.7%) patients underwent targeted therapy. During routine clinical follow-up appointments, the auxiliary examination, such as chest/brain MRI or CT, and blood samples were obtained. The median follow-up time for all patients was 33 months (range 0–135 months). During the follow-up, 3 patients died for heart failure and 12 matched blood samples were collected at the end of the first cycle of treatment. The RECIST version 1.1 (Eisenhauer et al. 2009) was used to evaluate treatment response, scoring responses as a complete response (CR), partial response (PR), progressive disease (PD) and stable disease (SD).Venous blood (5 mL) was collected into Ethylene Diamine Tetraacetic Acid (EDTA) anticoagulant tubes and processed within 4 h. This study was performed according to The Code of Ethics of the World Medical Association (Declaration of Helsinki) and approved by the Institutional Review Board (IRB) for human studies at the National Cancer Center/Cancer Hospital, Chinese Academy of Medical Sciences and Peking Union Medical College. The IRB number is 19/167-1951.

**Table 1:** Association between CTCs phenotype and clinicopathological features in 60 treatment naïve NSCLC

	*n*	Non-E/M-CTC						Hybrid E/M-CTC							
		E-CTC		M-CTC		Median	*P*	E > m-CTC		e = m-CTC		M > e-CTC		Median	*P*
		Median	*P*	Median	*P*			Median	*P*	Median	*P*	Median	*P*		
Age															
< 60	28	1 (0–17)	0.809	2 (0–24)	0.562	2.5 (0–29)	0.916	1 (0–17)	0.963	12.5 (0–65)	0.317	1 (0–15)	0.247	17.5 (0–83)	0.423
≥ 60	32	1 (0 ~ 11)		1 (0 ~ 47)		2.5 (0–48)		1 (0 ~ 8)		17.5 (0 ~ 58)		2 (0 ~ 21)		20 (0 ~ 68)	
Smoke															
No	37	0 (0–11)	0.120	1 (0–47)	0.183	1 (0–48)	0.102	0 (0–9)	0.013	11 (0–57)	0.059	1 (0–15)	0.812	16 (0–68)	0.045
Yes	20	1.5 (0–17)		2 (0–24)		4.5 (0–29)		2.5 (0–17)		22 (1–65)		1 (0–21)		27.5 (1–83)	
Unknown	3	0 (0–1)		1 (0–1)		1 (1–1)		0 (0–1)		10 (1–15)		1 (0–2)		12 (1–17)	
Gender															
Male	30	1 (0–17)	0.332	1 (0–24)	0.447	3 (0–29)	0.301	1 (0–17)	0.120	15.5 (0–65)	0.164	1 (0–21)	0.855	23 (0–83)	0.169
Female	30	0.5 (0–7)		1 (0–47)		1.5 (0–48)		0 (0–9)		11.5 (0–42)		2 (0–15)		16.5 (0–46)	
TNM stage															
I + II	31	0 (0–11)	0.206	1 (0–6)	0.007	1 (0–12)	0.013	1 (0–9)	0.518	18 (0–59)	0.169	2 (0–21)	0.885	22 (0–68)	0.137
III + IV	29	1 (0–17)		2 (0–47)		3 (0–48)		1 (0–17)		10 (1–65)		1 (0–9)		12 (1–83)	
Pathology															
Squamous carcinoma	8	1 (0–5)	0.846	2.5 (0–24)	0.194	3 (1–29)	0.165	1.5 (0–5)	0.900	15.5 (1–36)	0.913	2 (0–21)	0.188	19.5 (1–58)	0.974
Adenocarcinoma	52	1 (0–17)		1 (0–47)		1.5 (0–48)		1 (0–17)		15 (0–65)		1 (0–15)		18 (0–83)	
Differentiation															
Poor + Moderate	25	0 (0–11)	0.381	1 (0–4)	0.023	1 (0–12)	0.036	1 (0–9)	0.061	16 (0–59)	0.095	1 (0–6)	0.030	18 (0–63)	0.020
Well	8	1.5 (0–5)		1 (0–6)		2.5 (0–9)		4.5 (0–8)		33 (0–58)		3 (0–21)		41.5 (16–68–)	
Unknown	27	1 (0–17)		2 (0–47)		6 (0–48)		0 (0–17)		10 (1–65)		1 (0–9)		12 (1–83)	
Distant metastasis															
Positive	15	1 (0–17)	0.886	1 (0–47)	0.165	1 (0–48)	0.324	0 (0–6)	0.096	9 (0–28)	0.018	1 (0–6)	0.186	11 (0–34)	0.011
Negative	45	1 (0–11)		1 (0–13)		3 (0–13)		1 (0–17)		18 (0–65)		2 (0–21)		22 (0–83)	
Cyfra211 (0.0–3.3 ng/mL)															
> 3.3	19	1 (0–17)	0.750	1 (0–24)	0.291	2 (0–29)	0.682	2 (0–9)	0.412	15 (0–59)	0.696	1 (0–9)	0.661	25 (0–63)	0.972
≤ 3.3	25	1 (0–11)		1 (0–19)		2 (0–25)		1 (0–17)		18 (2–65)		1 (0–21)		22 (1–83)	
Unknown	16	0 (0–2)		1.5 (0–47)		3 (0–48)		0 (0–8)		9 (0–58)		1.5 (0–15)		12.5 (0–68)	
CEA (0.0–5.0 ng/mL)															
> 5.0	13	1 (0–6)	0.958	1 (0–19)	0.856	1 (0–25)	0.647	1 (0–17)	0.969	15 (0–65)	1.000	1 (0–9)	0.767	25 (0–83)	0.841
≤ 5.0	32	1 (0–17)		1 (0–24)		2.5 (0–29)		1 (0–9)		18 (1–36)		1 (0–21)		22 (1–58)	
Unknown	15	0 (0–2)		1 (0–47)		3 (0–48)		0 (0–8)		9 (0–58)		2 (0–15)		12 (0–68)	
SCC (0.0–1.5 ng/mL)															
> 1.5	6	1.5 (0–11)	0.881	0.5 (0–6)	0.628	2.5 (0–12)	0.777	3 (1–5)	0.061	25 (9–59)	0.193	1 (0–3)	0.907	31 (12–63)	0.170
≤ 1.5	38	1 (0–17)		1 (0–24)		1.5 (0–29)		1 (0–17)		16 (0–65)		1 (0–21)		21.5 (0–83)	
Unknown	16	0 (0–2)		1.5 (0–47)		3 (0–48)		0 (0–8)		9 (0–58)		1.5 (0–15)		12.5 (0–68)	

### Enrichment and identification of CTCs

CTCs enrichment was performed using the CanPatrol™ CTCs technique (SurExam, Guangzhou, China) as described previously (Xiang et al. 2018). First, lysis buffer was added to erythrocytes and then PBS containing 4% formaldehyde (Sigma-Aldrich) was used to resuspend the remaining cells for 5 min. Then, we transferred the cell suspension into a filtration tube and the pressure of the pump valve used was at least 0.08 MPa. The CanPatrol™ CTCs filtration system comprised a filtration tube containing a calibrated membrane (8-μm-diameter pores) (SurExam, Guangzhou, China), a manifold vacuum plate with valve settings (SurExam, Guangzhou, China), an E–Z 96 vacuum manifold (Omega, Norcross, USA) and a vacuum pump (Auto Science, Tianjin, China). The multiplex RNA in situ hybridization (RNA-ISH) assay was used to identify CTCs. Antibodies against the epithelial biomarkers CK8/18/19 and EpCAM, antibodies against the mesenchymal biomarkers vimentin and twist and the leukocyte biomarker CD45 were used to capture and characterize the CTCs. Nuclei were stained with 4',6-diamidino-2-phenylindole (DAPI). A fluorescent microscope were used to analyze all slides. An effective fluorescent dot was characterized to have at least 7 pixels. Positive signals presenting as punctate dots were quantified in each fluorescent channel.

### Measurement of tumor markers

A total of 3 mL of peripheral blood in tubes without anticoagulants (Becton Dickinson, Franklin Lakes, NJ, USA) was centrifuged at 1500*g* for 10 min. An automatic immunoassay analyzer (Cobas e601, Roche) was used to detect tumor markers, such as SCC, CEA and Cyfra 21-1.

### Statistical analysis

SPSS Statistics 25.0 software was used to analyze all data. For data not normally distributed, the differences between two groups was analyzed using the Mann–Whitney *U* test, while multi-group analysis was analyzed using the Kruskal–Wallis *H* test. To evaluate the diagnostic value of CTCs, a receiver operating characteristic (ROC) curve was established. To assess the predictive power, the area under the curve (AUC) was analyzed. A two-tailed *P* value of < 0.05 was considered statistically significant. Graphs were plotted using GraphPad Prism 5.

## Results

### CTCs detection in the peripheral blood of NSCLC patients

**Fig. 1:**
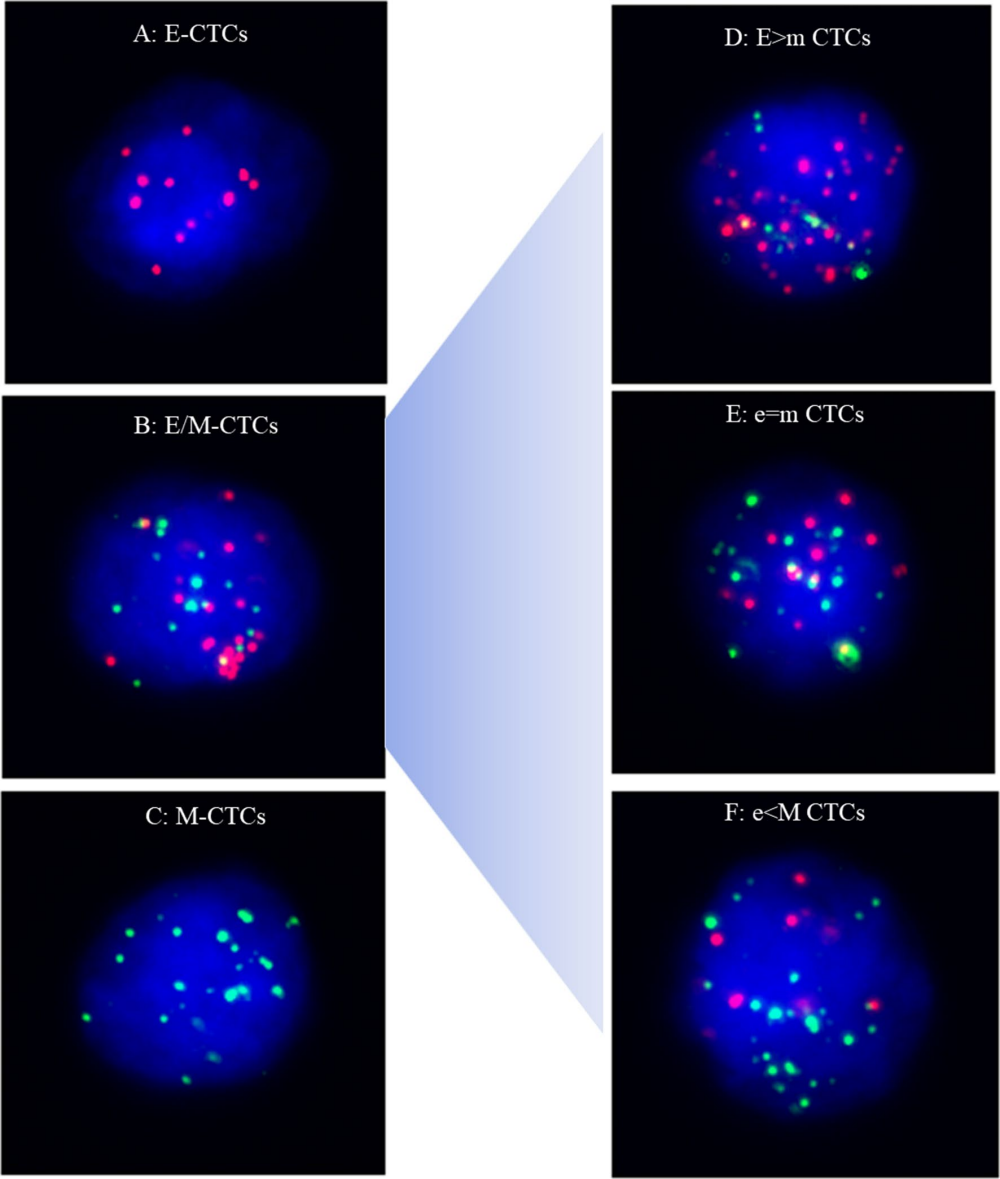
Representative images of five types of CTCs isolated from NSCLC patients based on RNA-ISH of epithelial marker(red dots) and mesenchymal marker (green dots). **A** E-CTCs: epithelial marker + , mesenchymal marker-, CD45–, and DAPI + cells; **B**: hybrid E/M-CTCs: epithelial marker + , mesenchymal marker + , CD45–, and DAPI + cells; **C** M-CTCs: epithelial marker-, mesenchymal marker + , CD45–, and DAPI + cells; **D** E > m CTCs (the fluorescence dot counts of epithelial markers were double compared to the mesenchymal markers); **E** e = m CTCs (the fluorescence dot counts of epithelial markers were similar to mesenchymal markers); **F** e < M CTCs (the fluorescence dot counts of epithelial markers were two times lower than the mesenchymal markers)

**Fig. 2:**
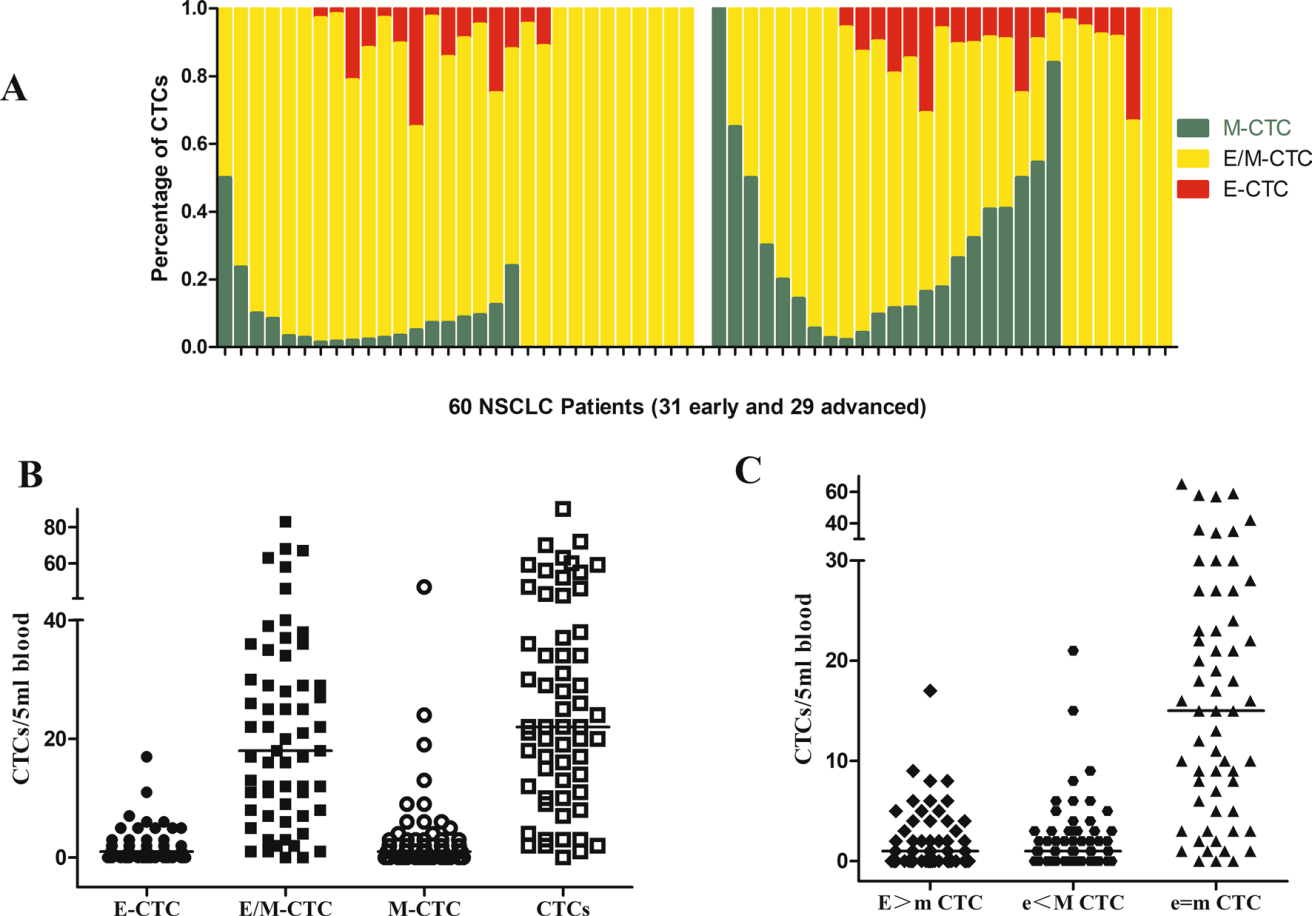
CTCs phenotypes in 60 treatment naïve NSCLC; **A** Percentage of E-CTCs, hybrid E/M-CTCs, M-CTCs for each patients (31 early stage and 29 advanced); **B** Distribution of total CTCs, E-CTCs, hybrid E/M-CTCs, M-CTCs in 60 NSCLC patients; **C** Distribution of E > m CTCs, e = m CTCs, e < M CTCs in 60 NSCLC patients

**Fig. 3:**
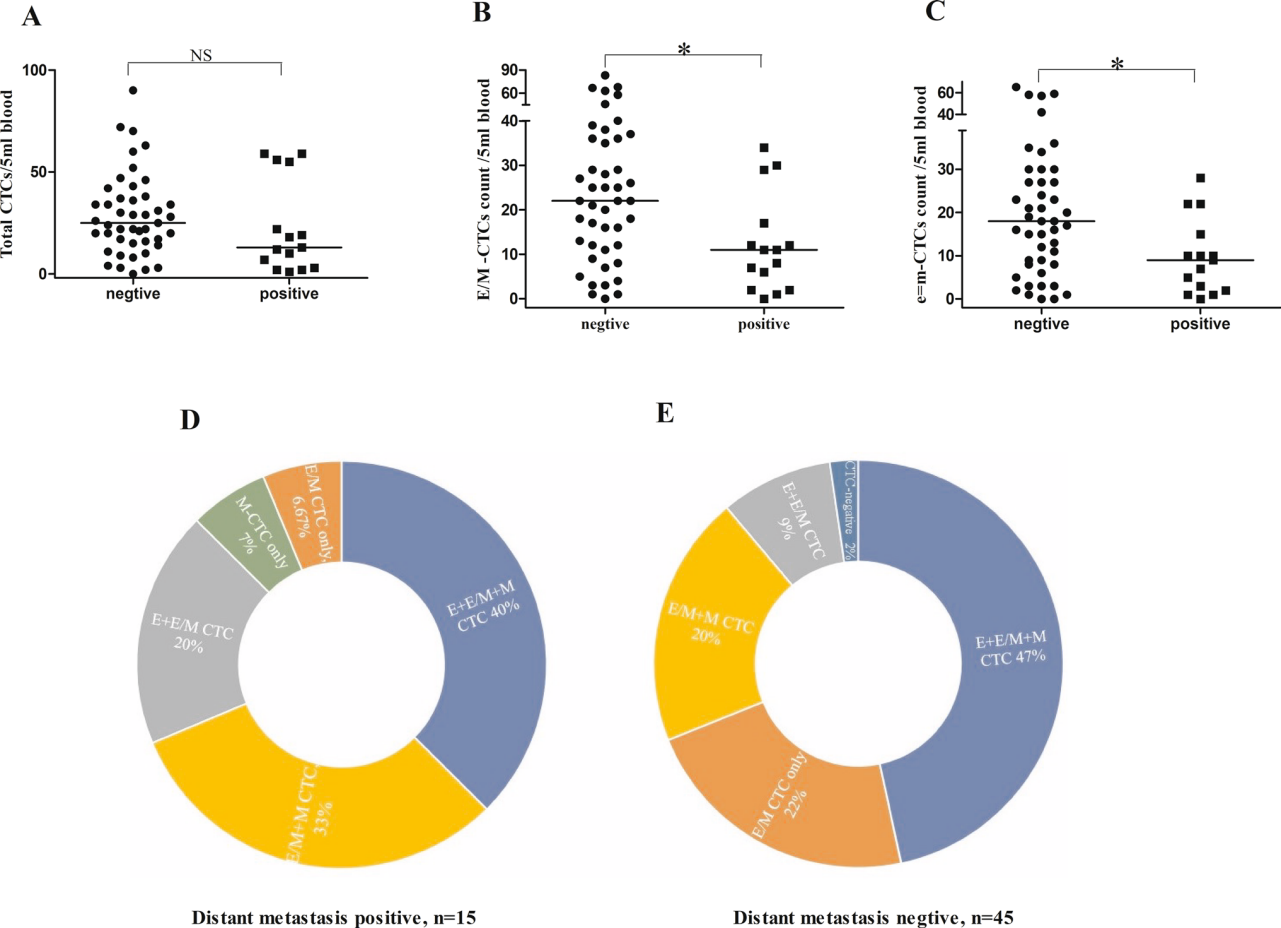
CTCs phenotypes and distant metastasis; Distribution of total CTCs (**A**), E/M CTCs (**B**), e = m-CTCs (**C**) between patients with and without distant metastasis; (**D**) 15 distant metastasis positive patients were further analyzed. One out of fifteen patients (7%) with distant metastasis was detected pure hybrid E/M-CTCs, and pure M-CTC; 3/15(20%) were detected both E-CTCs and E/M-CTCs (E + E/M CTCs); 5/15(33%) were detected both E/M CTCs and M-CTCs (E/M + M CTCs); 6/15(40%) detected the three phenotypes (E + E/M + M CTCs); **E** 45 patients without distant metastasis were analyzed, 10/45(22%) were detected pure hybrid E/M CTCs; 4/45(9%) were detected E + E/M CTCs, 9/45(20%) were detected E/M + M CTCs, 21/45(47%) were detected E + E/M + M CTCs, 1 was detected none CTCs

Quantification of EMT states in each CTC were based on dot counts of epithelial and mesenchymal markers. CTCs were classified into three phenotypes, including (1) E-CTCs: epithelial marker + , mesenchymal marker-, CD45−, and DAPI + cells (Fig. 1A); (2) hybrid E/M-CTCs: epithelial marker + , mesenchymal marker + , CD45–, and DAPI + cells (Fig. 1B); (3) M-CTCs: epithelial marker-, mesenchymal marker + , CD45−, and DAPI + cells (Fig. 1C). Leukocytes were identified as CD45 + and DAPI + cells. Compared to hybrid E/M-CTCs, E-CTCs and M-CTCs were referred to as non-E/M CTCs. Moreover, according to the fluorescence dot counts of epithelial and mesenchymal markers, hybrid E/M phenotype CTCs were further divided into three subtypes, including E > m CTCs (the fluorescence dot counts of epithelial markers were double compared to the mesenchymal markers) (Fig. 1D), e = m CTCs (the fluorescence dot counts of epithelial markers were similar to mesenchymal markers) (Fig. 1E)and e < M CTCs (the fluorescence dot counts of epithelial markers were two times lower than the mesenchymal markers) (Fig. 1F).

In 82 NSCLC cohort, only 2 patients didn’t detect CTCs, the overall CTCs detection rate was 97.5% (80/82). The median number of total CTCs per 5 mL blood was 21 (range 0–90). Across all the patients, the median number of hybrid E/M phenotype CTCs was 17 cells/5 mL (range 0–83), and the median number of E-CTCs and M-CTCs was 1 cells/5 mL (range 0–17 and 0–47, respectively).

For 60 treatment naïve NSCLC, only one patient didn’t detect CTCs and 27 patients showed all three phenotypes. A total of 13 patients showed both M-CTCs and hybrid E/M-CTCs, 7 patients showed both E-CTCs and hybrid E/M-CTCs, 11 patients showed pure E/M-CTCs and 1 patient showed pure M-CTCs (Fig. 2A).

The total number of CTCs detected in the 59 treatment naïve NSCLC ranged from 1 to 90 cells/5 mL with the median value being 22 cells/5 mL. The number of hybrid E/M phenotype CTCs, the most common phenotype observed in each patient, ranged from 0 to 83 cells/5 mL with a median value of 13 cells/5 mL. The number of E-CTCs and M-CTCs ranged from 0 to 17 cells/5 mL and 0 to 47 cells/5 mL, respectively, with the median value being 1 cells/5 mL (Fig. 2B).

According to the fluorescence intensity of epithelial and mesenchymal markers, hybrid E/M phenotype CTCs were further divided into three subtypes, including the number of E > m CTCs, e = m CTCs and e < M CTCs that contained ranges of 0–17 cells/5 mL, 0–65 cells/5 mL and 0–21 cells/5 mL, with median values being 1 cells/5 mL, 15 cells/5 mL and 1 cells/5 mL, respectively (Fig. 2C).

### Correlation between CTCs phenotype and clinicopathological characteristics

The relationship between CTCs and clinicopathological characteristics in 60 treatment naïve NSCLC is shown in Table 1. Hybrid E/M-CTCs counts significantly differed between patients that were smokers vs non-smokers, well-differentiated vs poor or moderate differentiated NSCLC, with vs without distant metastasis. The non-E/M CTCs, especially the M-CTCs, significantly differed between early and advanced stage patients. No significant differences were observed when analyzing gender, pathological type and tumor markers, such as Cyfra211, CEA and SCC.

The distribution of total CTCs/5 ml blood between advanced vs early stages showed no significant differences (Supplementary Fig. 1A). However, the non-E/M CTCs, especially the M-CTCs, were significantly greater more in advanced stage NSCLC patients compared to patients in early stages (Supplementary Fig. 1B, C). The ROC curve with a cutoff value of M-CTCs being 2 cells/5 mL blood demonstrated that the sensitivity of M-CTCs in the diagnosis of advanced NSCLC was 58.62% and the specificity was 77.42% (AUC = 0.6974, 95% CI 0.5609–0.8340). (Supplementary Fig. 1D).

The distribution of total CTCs/5 ml blood between patients with vs without distant metastasis showed no significant difference (Fig. 3A), while hybrid E/M CTCs, especially the e = m-CTCs, significantly differed between them(Fig. 3B, C). Interestingly, compared to distant metastasis cohort, patients without distant metastasis had more number of hybrid E/M CTCs and e = m-CTCs.

Findings showed that 1/15patients (7%) with distant metastasis showed pure hybrid E/M-CTCs and pure M-CTC, a total of 3/15 (20%) showed both E-CTCs and E/M-CTCs (E + E/M CTCs), a total of 5/15 (33%) showed both E/M CTCs and M-CTCs (E/M + M CTCs) and a total of 6/15 (40%) showed three phenotypes (E + E/M + M CTCs). In patients without distant metastasis, a total of 10/45 (22%) showed pure hybrid E/M CTCs, a total of 4/45 (9%) showed E + E/M CTCs, a total of 9/45 (20%) showed E/M + M CTCs, a total of 21/45 (47%) showed E + E/M + M CTCs and only one did not show detectable levels of CTCs (Fig. 3D, E). Though statistical analysis showed that the distribution of CTCs subpopulations in patients with and without distant metastasis was not statistically significant, from the figures, we found that patients with pure hybrid E/M-CTCs showed a lower proportion in distant metastasis positive cohort compared to negative ones(7% vs 22%), while patients with E + E/M CTCs (20% vs 9%) and E/M + M CTCs (33% vs 20%) showed a higher proportion. 7% distant metastasis positive cohort showed M-CTCs phenotype, and no one patients for distant metastasis negative cohort.

### Correlations between CTCs phenotype and tumor size

A total of 27 patients were diagnosed in early stage without regional lymph node and distant metastasis. Among these patients, the correlation between CTC phenotypes and tumor size were further analyzed. Tumor size was defined as ﻿the maximum diameters of target lesions which was resected and measured by pathologist. The Spearman correlation showed that e = m CTCs slightly correlated with tumor size. The higher number of CTCs, the larger the tumor size (Supplementary Fig. 2).

### Dynamic changes of CTCs in advanced NSCLC patients

The CTCs count was determined in 12 patients prior to therapy as well as after targeted therapy or radiotherapy.

A total of 6 patients with stage IV received targeted therapy, since they had EGFR mutation or ALK fusion. After 1 month, 1 patient (case 1) measured as PD showed both decreased E-CTCs and E/M-CTCs but increased M-CTCs. This patient had bone metastasis after 3 months. Two patients (case 2 and case 5) measured as SD showing decreased number of all three CTCs (E-CTCs, E/M-CTCs and M-CTCs). Case 4 measured as PD showed decreased E/M-CTCs but increased E-CTCs and M-CTCs with a stable primary tumor lesion that enlarged 10 months later and was. In the last two cases (case 3 and case 6) measured as PD, E-CTCs, E/M-CTCs and M-CTCs were elevated (Supplementary Fig. 3).

The other 6 patients diagnosed with stage III disease received concurrent chemoradiation. After one cycle treatment, four patients (case 7, case 10, case 11 and case 12) with decreased or no significant changes in the number of E-CTCs, E/M-CTCs, M-CTCs showed tumor reduction and was measured as SD. One patient (Case 8), that contained both elevated E/M-CTCs and M-CTCs showed lymph node metastasis and was measured as PD. One case (Case 9) contained decreased E-CTCs and E/M-CTCs but increased M-CTCs showed SD. These data suggested that E/M-CTCs were associated with primary tumor size, while M-CTCs were associated with disease progression (Supplementary Fig. 4).

## Discussion

CTCs are an independent prognostic indicator of progression-free survival and overall survival in advanced NSCLC(Lindsay CR et al. 2019), pancreatic cancer (Zhang et al. 2015) and prostate cancer (Heller et al. 2018).

EMT plays an important role in tumor metastasis (Reduzzi et al. 2020; Tayoun et al. 2019). Cells lose their specific epithelial characteristics during EMT, such as apicobasal polarity, intercellular adhesion complexes and cytoskeletal architecture (Guo et al. 2019). They then acquire mesenchymal characteristics, such as cell polarity, migration ability and invasive ability (Tulchinsky et al. 2019). Previous found that CTCs increased metastasis and invasion through EMT in breas t(Yu et al. 2013; Zhang et al. 2017) and lung (Tulchinsky et al. 2019; Zhang et al. 2019) cancer patients. This indicated that the CTC mesenchymal phenotype was an important biomarker for disease progression or target-therapy resistance. However, others found that EMT was dispensable for metastasis in pancreatic cancer (Zheng et al. 2015). Here, we analyzed the heterogeneous phenotypes in NSCLC and try to find which phenotype CTCs are associated with metastasis in therapy-naïve NSCLC patients.

Using Canpatrol™ CTC assays, CTCs were classified into three phenotypes, including E-CTCs, hybrid E/M-CTCs and M-CTCs. Hybrid E/M phenotype CTCs were further divided into three groups, including E > m CTCs, e = m CTCs and e < M CTCs. We found that the hybrid E/M phenotype CTCs, especially e = m CTCs, were the most common phenotype observed in each patient and this phenotype is a highly heterogeneous hybrid. This is consistent with the putative theory that most tumor cells do not undergo terminal differentiation but remain in intermediate stages (Tulchinsky et al. 2019). Hybrid E/M phenotype CTCs were also related to the primary tumor size. It was speculated that these tumor cells had a strong ability to proliferate and survive after obtaining the mesenchymal phenotype. M-CTCs can be used to distinguish the early and late stages of disease, indicating that the intravasation and extravasation ability is enhanced after acquiring the mesenchymal phenotype.

Zhang et al. (2019) found that E + /M + CTCs could be used to distinguish NSCLC from benign pulmonary diseases, while M + CTCs could differentiate distant metastasis vs non‑distant metastasis NSCLC patients. In our study, we found that patients containing pure hybrid E/M-CTCs were less likely to have distant metastasis compared to patients containing both E-CTCs and E/M-CTCs, indicating that the occurrence of metastasis in the microenvironment requires the cooperation among different subtype s(Agnoletto et al. 2019). Moreover, recent studies support our results, revealing that epithelial phenotype CTCs were associated with lung metastases and these CTCs also showed a restricted mesenchymal transition (Williams et al. 2019). Whereas pure mesenchymal CTCs were less likely to be associated with metastasis (Liu et al. 2019).

Tumor metastasis theory of EMT-MET (Bakir et al. 2020) believes that cell phenotype can change continuously and dynamically during the process of tumor formation and metastasis. EMT occurs in early stage of disease progression, and there are different EMT states in the process of metastasis cascade. EMT, where tumor cells lose their epithelial characteristics and acquire mesenchymal characteristics, represents a salient property of primary tumor formation and progression. MET is the reverse process of EMT and is apparent during tumor metastasis. Cells re-express epithelial features, such as EpCAM or Ecad, at the metastatic site. Research in SCLC (Hamilton and Rath 2017) thought that MET occurred in the peripheral blood prior to metastasis colonization. Expression of epithelial characteristics at a metastatic site is employed as possible evidence for MET. Using a lineage labeling method to track dissemination in a spontaneous metastasis model of pancreatic cancer (Aiello et al. 2016), they found that epithelial markers such as Ecad and Claudin-7 increased as disseminated cells evolved from isolated tumor cells to micrometastic and macrometastatic clusters, while mesenchymal markers decreased. The larger the liver metastasis, the more it recapitulates the epithelial characteristics of the primary tumor. Based on the hypothesis of EMT-MET tumor metastasis, expression of epithelial may predict the possibility of distant metastasis, that’s may be why E + E/M CTCs are more closely related to distant metastasis than pure E/M CTCs.

In this study, the diagnosis of patients with distant metastasis is also based on imaging. A retrospective analysis of 60 treatment naïve NSCLC found that the distribution of hybrid E/M-CTCs between patients with or without distant metastasis was statistically significant. In 12 matched patients, after receiving a cycle of treatment, treatment evaluation and CTCs subgroup analysis also indicated that E/M-CTCs were related to the size of the primary tumor, and the increase of M-CTCs might indicate disease progression, especially Case1 and Case 4, whose imaging showed disease progression at 3 months and 10 months, respectively. In theory, CTC can spread into blood at very early stage of the disease (Bakir et al. 2020), using CTC profile to predict distant metastasis could be earlier than imaging examinations, especially for tumor micrometastasis.

However, the current low sensitivity of CTC detection technology and low enrichment of the cells that underwent EMT limited their clinical application. Combination of multiple surface markers and more effective methods to isolate single cells, such as single-cell sequencing technologies, will help pave the way for these clinical transformations. Moreover, due to our small number of samples, the statistical results may have a certain degree of statistical error. A larger population is needed to be further verified.

In conclusion, heterogeneous CTCs exists in the peripheral blood of NSCLC patients, and epithelial features with a hybrid epithelial–mesenchymal phenotype are associated with metastasis in therapy-naïve patients. Further studies are still needed to investigate CTC patterns and their clinical translation.

## Supplementary Information

Below is the link to the electronic supplementary material.

Supplementary Figure 1 CTCs phenotypes and TNM stage; Distribution of total CTCs (A), non-E/M CTCs (B), M-CTCs (C) between early stage (I+II) and advanced stage (III+IV) NSCLC patients; (D) ROC curve demonstrated that when the cutoff value of M-CTCs was 2 cells/5 mL blood, the sensitivity of M-CTCs in the diagnosis of advanced NSCLC was 58.62%, and the specificity was 77.42% (AUC=0.6974, 95% CI, 0.5609 to 0.8340). NS: non-significance; *p < 0.05

Supplementary Figure 2 Correlations between CTCs phenotype and tumor size

Supplementary Figure 3 CTCs dynamic change monitoring in 6 stage IV patients before and after targeted therapy

Supplementary Figure 4 CTCs dynamic change monitoring in 6 stage III patients before and after radiotherapy therapy

## Data Availability

All data generated or analyzed during this study are included in this published article [and its supplementary information files].

## Code availability

All data and code generated or used during the study appear in the submitted article.
