# Supplementary figures and images for: Epithelial circulating tumor cells with a heterogeneous phenotype are associated with metastasis in NSCLC
Source: J Cancer Res Clin Oncol. 2021 Jul 13;148(5):1137–46. doi: 10.1007/s00432-021-03681-9 (PMC9016037; doi:10.1007/s00432-021-03681-9)

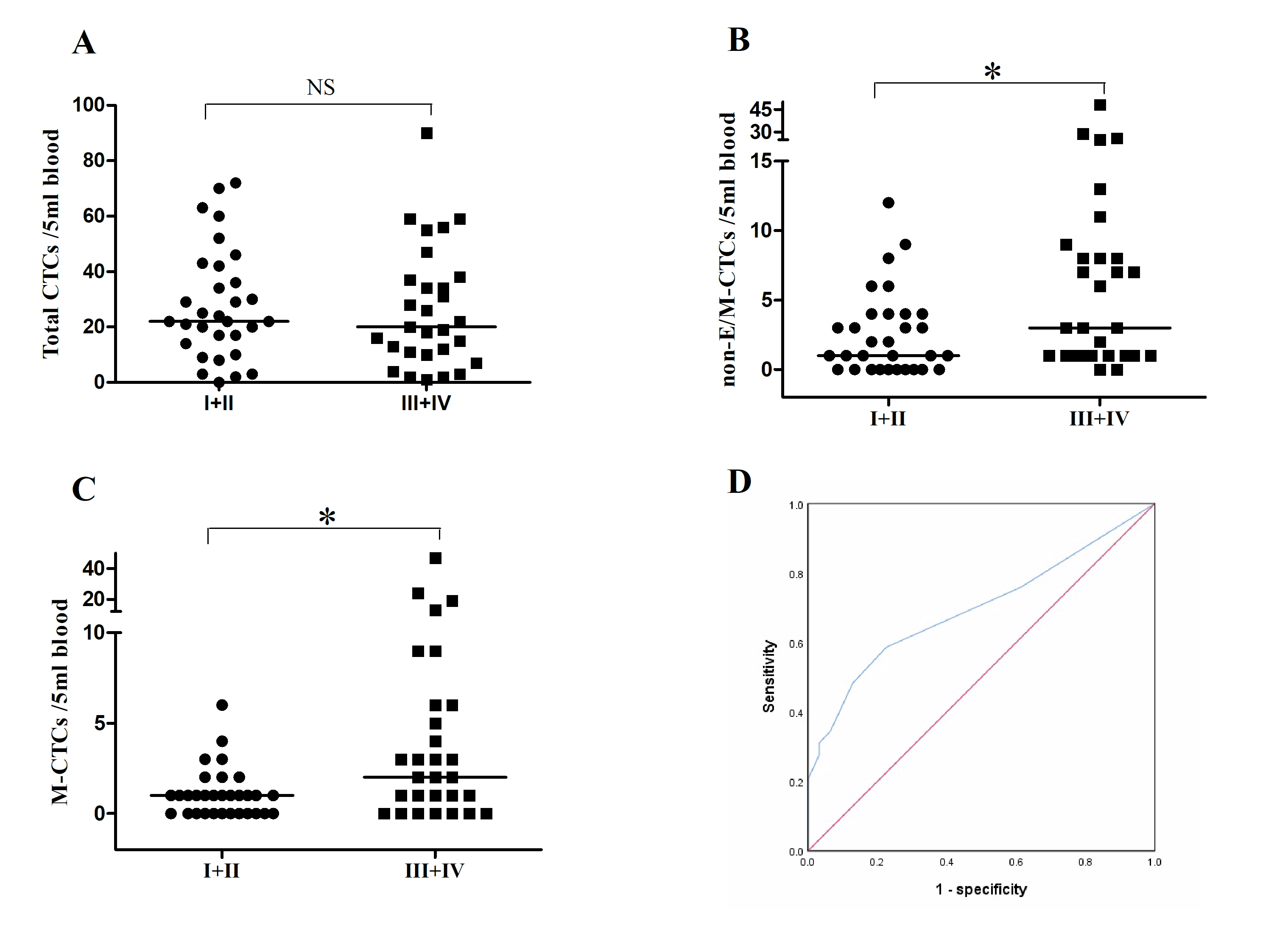

Supplement: Supplementary file 1 — Supplementary Figure 1 CTCs phenotypes and TNM stage; Distribution of total CTCs (A), non-E/M CTCs (B), M-CTCs (C) between early stage (I+II) and advanced stage (III+IV) NSCLC patients; (D) ROC curve demonstrated that when the cutoff value of M-CTCs was 2 cells/5 mL blood, the sensitivity of M-CTCs in the diagnosis of advanced NSCLC was 58.62%, and the specificity was 77.42% (AUC=0.6974, 95% CI, 0.5609 to 0.8340). NS: non-significance; *p < 0.05 [file 432_2021_3681_MOESM1_ESM.tif]

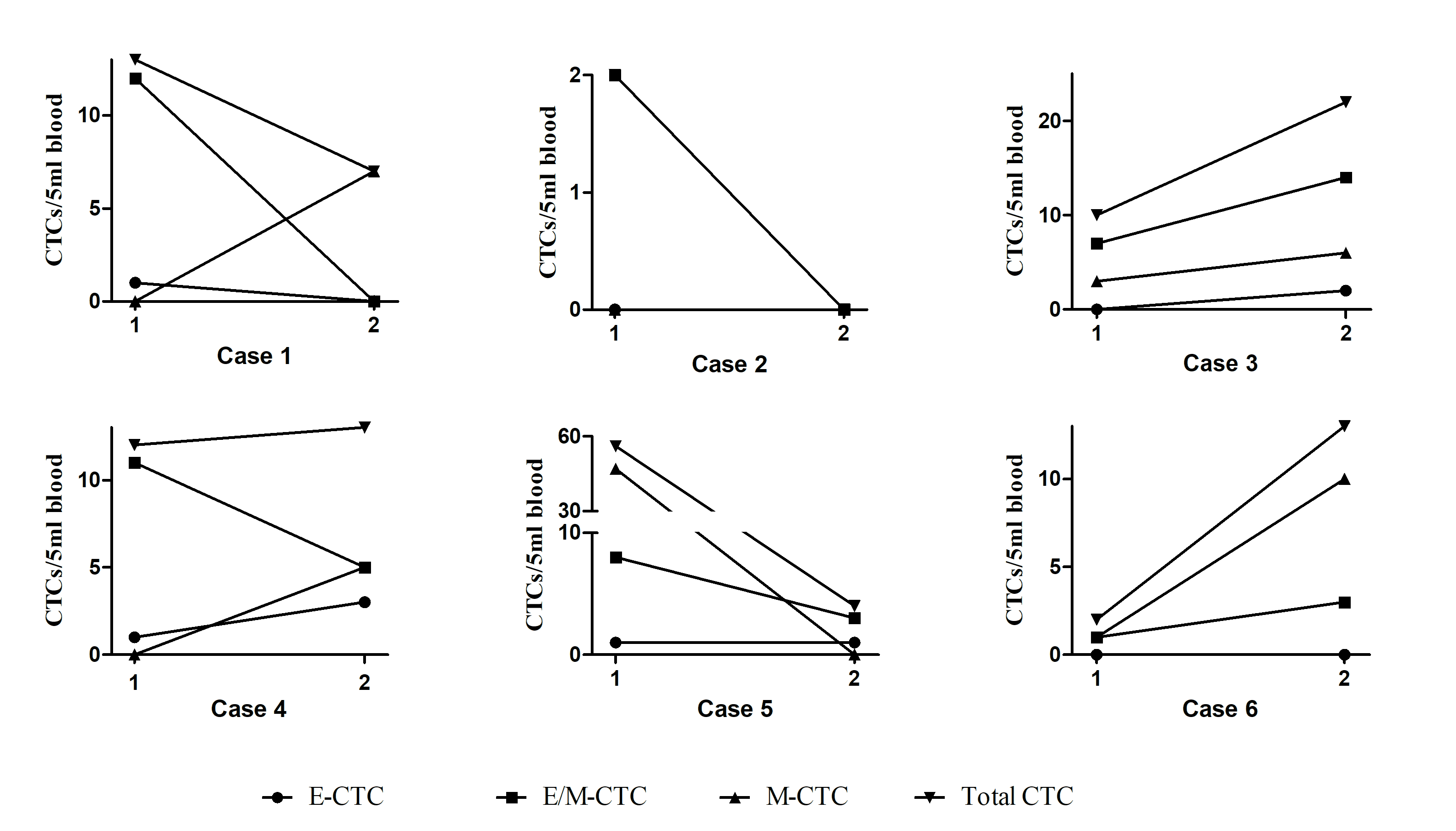

Supplement: Supplementary file 2 — Supplementary Figure 2 Correlations between CTCs phenotype and tumor size [file 432_2021_3681_MOESM2_ESM.tif]

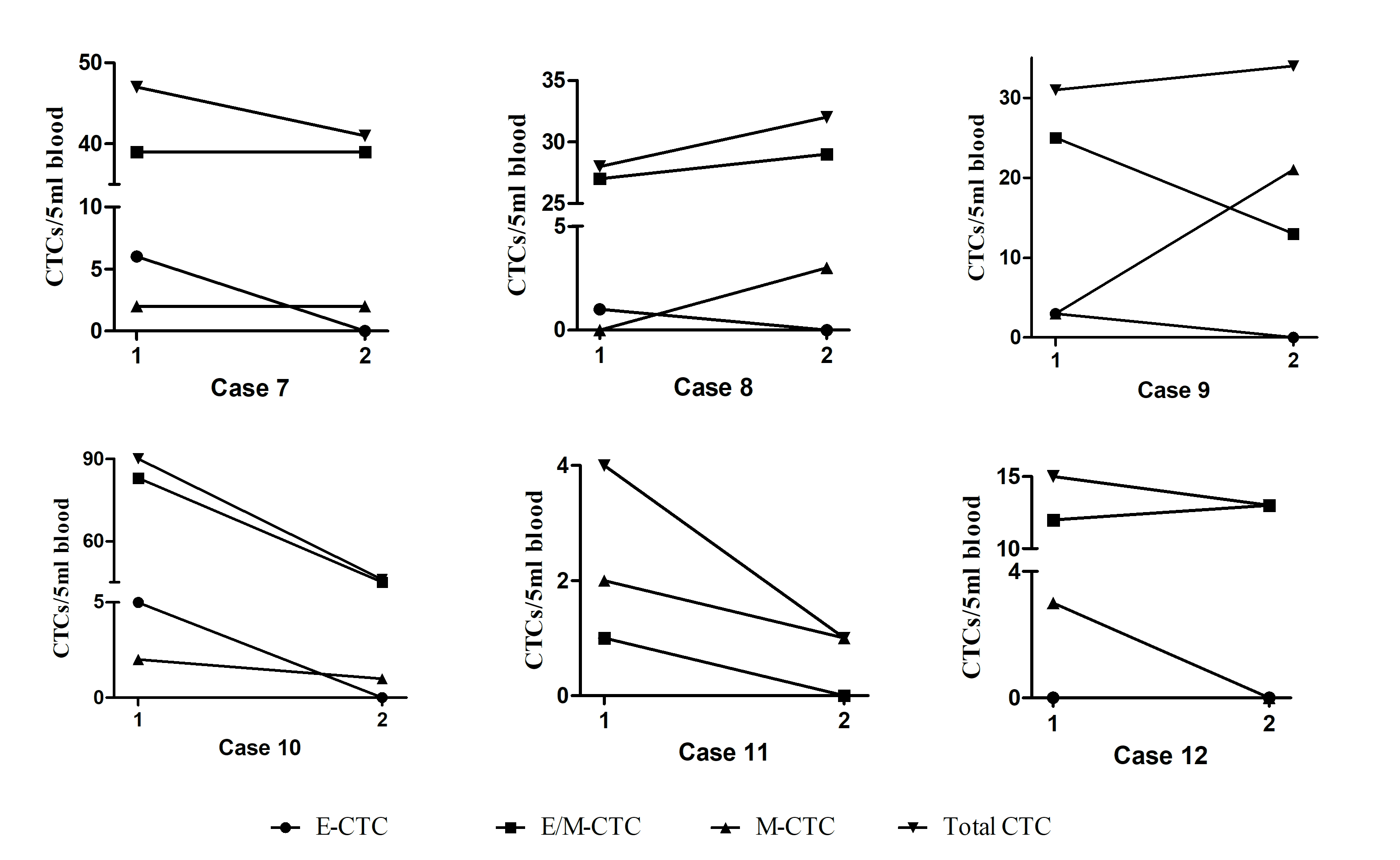

Supplement: Supplementary file 3 — Supplementary Figure 3 CTCs dynamic change monitoring in 6 stage IV patients before and after targeted therapy [file 432_2021_3681_MOESM3_ESM.tif]

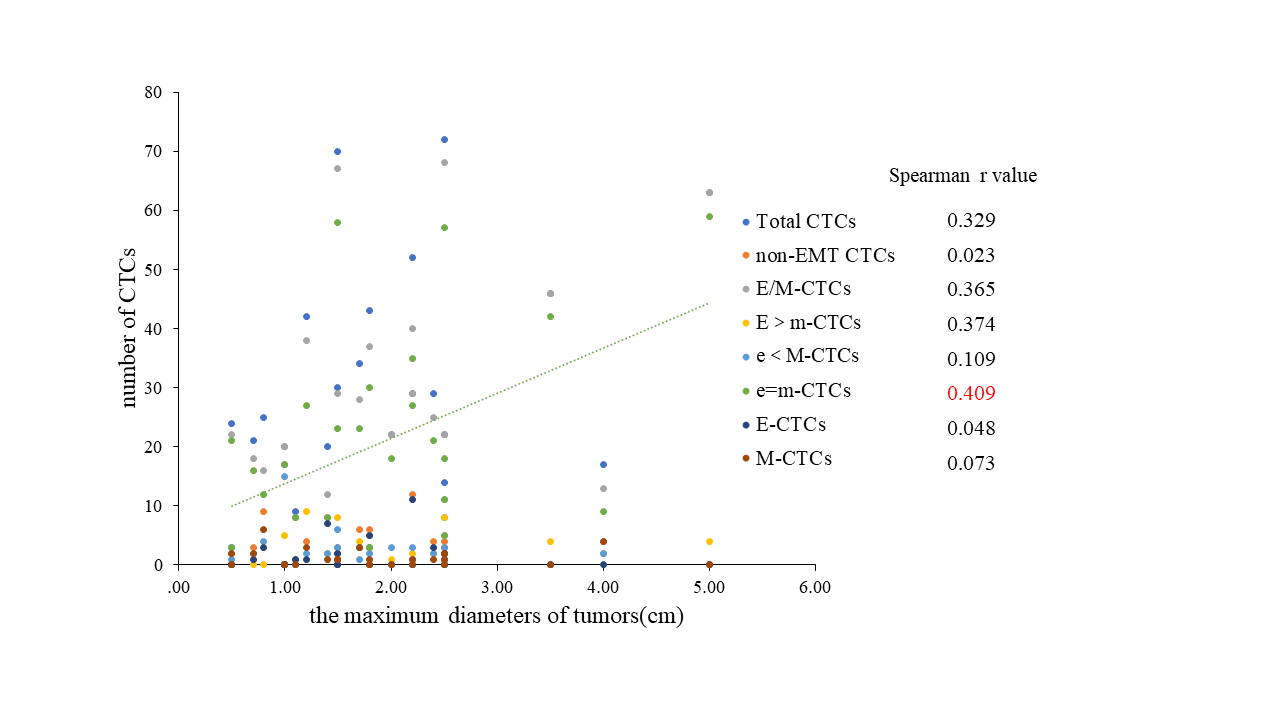

Supplement: Supplementary file 4 — Supplementary Figure 4 CTCs dynamic change monitoring in 6 stage III patients before and after radiotherapy therapy [file 432_2021_3681_MOESM4_ESM.tif]
